# Integrating omics reveals insights into tomato abaxial/adaxial leafy supplemental lighting

**DOI:** 10.3389/fpls.2023.1118895

**Published:** 2023-04-05

**Authors:** Chengyao Jiang, Haolian Wu, Xiaoying Zhang, Jiaming Liu, Yushan Li, Yu Song, Jue Wang, Yangxia Zheng

**Affiliations:** ^1^ College of Horticulture, Sichuan Agricultural University, Chengdu, China; ^2^ Laboratory of Crop Immune Gene Editing Technology, Chengdu NewSun Crop Science Co., Ltd., Chengdu, China; ^3^ Research Institute of Crop Germplasm Resources, Xinjiang Academy of Agricultural Sciences, Urumqi, China

**Keywords:** *Solanum lycopersicum*, irradiation orientation, photosynthesis, transcriptomic, proteomic

## Abstract

Research revealed that the abaxial leafy supplemental lighting (AB) can significantly improve the net photosynthetic rate and stomatal conductance in the leaves of tomato plants compare to the adaxial leafy supplemental lighting (AD) method. However, the underlying regulatory mechanisms are still poorly understood. Here, we conducted AB and AD on tomato and assessed transcriptomic, and proteomic changes in leaves. The result showed that under the two supplemental lighting methods, a total of 7352 genes and 152 proteins were differentially expressed. Significant differences were observed in genes expression levels and proteins abundances across multiple pathways, mainly including cell process, metabolism process, biological regulation, environment information processing, genetic information processing, metabolism, and organismal systems. Additionally, we also found that some key genes that plant hormone signaling, light perception, photosynthesis, plant fitness, and promoting fruit ripening, have increased significantly, which can explain the effect of AB on plant growth and development. Finally, through the qPCR, we determined that AB mainly up-regulate a series of auxin-responsive genes or factors, auxin polarity transport genes, gibberellin synthesis genes, cell cycle regulator genes, sugar transporters, and fleshy fruit ripening genes. These results help us to understand plant light response mechanism and discover genes which contribute to efficient light energy utilization.

## 1 Introduction

Photosynthesis plays a crucial role in determining plant yield, with 90% to 95% of the dry weight of the crop coming from this process (Murchie et al., 2009; Simkin et al., 2020). Improving photosynthetic efficiency can increase crop yield by over 50% (Covshoff and Hibberd, 2012). In tomato production, even a 1% increase in light irradiation can lead to a 20% increase in leaf photosynthesis, resulting in an over 1% increase in fruit yield and improved fruit quality (Jiang et al., 2017; Geelen, 2018). Therefore, enhancing photosynthesis has always been a research priority for improving tomato yield and quality.

Tomatoes (*Solanum lycopersicum*) are nutritious and widely cultivated in the world. In China, even one-third of greenhouses are used for tomato production. This plant prefers sunlight and warm climate. However, in Northern China, greenhouse tomato production faces a challenge of light insufficiency due to greenhouse shading, continuous rainy or snowy weather in winter and spring, and intensive cultivation schedules, greenhouse tomato production which can cause growth failure, decreased fruit yield and quality, and ultimately reduced profitability (Acock et al., 1978; Xu et al., 1997; Hogewoning et al., 2010; Lu et al., 2012; Terfa et al., 2013; Tewolde et al., 2016). Improving the light environment and enhancing the utilization of light energy by plants have become critically important in greenhouse tomato production.

Artificial supplemental lighting can improve the light conditions of plant canopies. Numerous studies have focused on the canopy layer (Hovi et al., 2004; Hovi and Tahvonen, 2008; Pettersen et al., 2010), light source (Lu et al., 2012; Song et al., 2016), light intensity (Dorais, 2003; Song, 2017), and light period (Matsuda et al., 2014; Tewolde et al., 2016). Recent studies have reported exciting results regarding the effective positioning of supplemental lighting (Zhang et al., 2015; Song et al., 2016; Zhang et al., 2016; Jiang et al., 2017; Li et al., 2017; Song, 2017; Jiang et al., 2019; Song et al., 2021; Jiang et al., 2022), including the use of abaxial leafy supplemental lighting (AB) and adaxial leafy supplemental lighting (AD) to greenhouse tomatoes. Leaves irradiated with AB demonstrated a 15.8% increase in the quantum yield of PSII electron transport (ФPSII) compared to those treated with AD, resulting in a tomato fruit yield increase of at least 10.7% (Song et al., 2016; Jiang et al., 2017; Jiang et al., 2022). Similar results were observed in grapes (Li et al., 2017), and lettuce (Zhang et al., 2015). Blue light irradiation of grape leaf abaxial surfaces significantly increased CO_2_ assimilation, while compound and red light both enhanced berry mass (Li et al., 2017), suggesting that light spectrum wavelength can have composite effects. Moreover, research on the characterization of photosynthetic gas exchange in leaves of trees (*Platanus orientalis* L. and *Melia azedarach* L.) and herbs (*Solanum lycopersicum* L.) demonstrated that bifacial leaves can fix more carbon than leaves with one irradiation surface when exposed to the same irradiation amount (Zhang et al., 2016). These exciting findings inspired us to consider efficient irradiated surfaces of the functional blade as a method to enhance the profitability of supplemental lighting in greenhouses. Additionally, these findings piqued our curiosity regarding the underlying mechanism which may present a new starting point for overcoming the damage of light insufficiency stress to greenhouse vegetable production and ensuring both high yield and quality of greenhouse-grown tomatoes.

The developmental mechanism that governs the functional behavior and formation of flat leaf lamina in relation to adaxial–abaxial fate has long been of interest to biologists. For several decades, researchers have recognized a functional relationship between photosynthesis activity and the differentiation of adaxial and abaxial leaf fate. According to some scholars the thicker cuticle of the leaf surface and smaller chloroplast volume lead to the highest internal photosynthesis rates in the middle and lower palisade layers, rather than near the adaxial leaf surface (Sun and Nishio, 2001; Evans and Vogelmann, 2003; Soares et al., 2008). Moreover, the adaxial/abaxial specification in the regulation of photosynthesis is influenced by the differential sensitivity of stomatal opening to light orientation and fixed gradients of enzyme activation across the leaf (Soares et al., 2008). Although several distinct regulators involved in leaf adaxial–abaxial photosynthetic response and lamina outgrowth have been identified (Yamaguchi et al., 2012), the underlying regulatory mechanisms, are still insufficiently understood, and the molecular basis of this interaction remains unclear.

In this study, we treated greenhouse-grown tomatoes with both AB and AD and assessed transcriptomic and proteomic changes in the leaves. By analyzing the significant differences in genes expression levels and proteins abundances, we hope to locate key genes and possible pathways involved, and create a regulatory map that we can use to investigate the underlying regulatory mechanisms of AB on tomatoes. This study provides useful knowledge for improving both the light-use efficiency of plants and fruit yield by adjusting artificial light sources.

## 2 Materials and methods

### 2.1 Materials and plant growth condition

Tomato (*S. lycopersicum*) ‘*Jinpeng* No.1’(Ding et al., 2019) was used in this research and experiments were conducted in Chengdu, Sichuan Province, China (104.06°E, 30.67°N) between December 2021 and March 2022. Seeds were sown in a plastic seedling tray (53 × 27.5 × 4.5 cm) filled with substrate (Pindstrup, Demark) and housed within an artificial climate chamber (RTOP-1000D, Top Yun Co. Ltd., Hanzhou, China) with climate settings held at 28 ± 1°C during the day and 18 ± 1°C during the night with 65 ± 5% relative humidity and a photoperiod of 14 h. Three replicated groups, each containing 100 seeds were established. Three weeks after sowing, 60 uniform seedlings from each group with two fully expanded leaves were transplanted into 7 × 7 × 8 cm black plastic pots filled with substrate (Pindstrup, Demark) in a Venlo-type arrangement, with double spans in a north–south orientation greenhouse (9.6×4×4.5m) at a set climate of 30°C/15°C temperatures (day/night) and 65% relative humidity with automatic air conditioning. The daily maximum natural indoor light intensity (PPFD) varied from 100 to 250 µmol·m^-2^·s^-1^ (measured at the same height of top canopy of tomato plants). Plants were set 10-13 cm apart from one another. After three weeks of irrigation with a half dose of Yamazaki nutrient solution (EC 1.0 ± 0.2 mS/cm), the dose of the solution was doubled (pH 6.5 ± 0.5, EC 2.0 ± 0.5 mS/cm) until the conclusion of the experiment.

### 2.2 Supplemental lighting treatment

**Figure 1:**
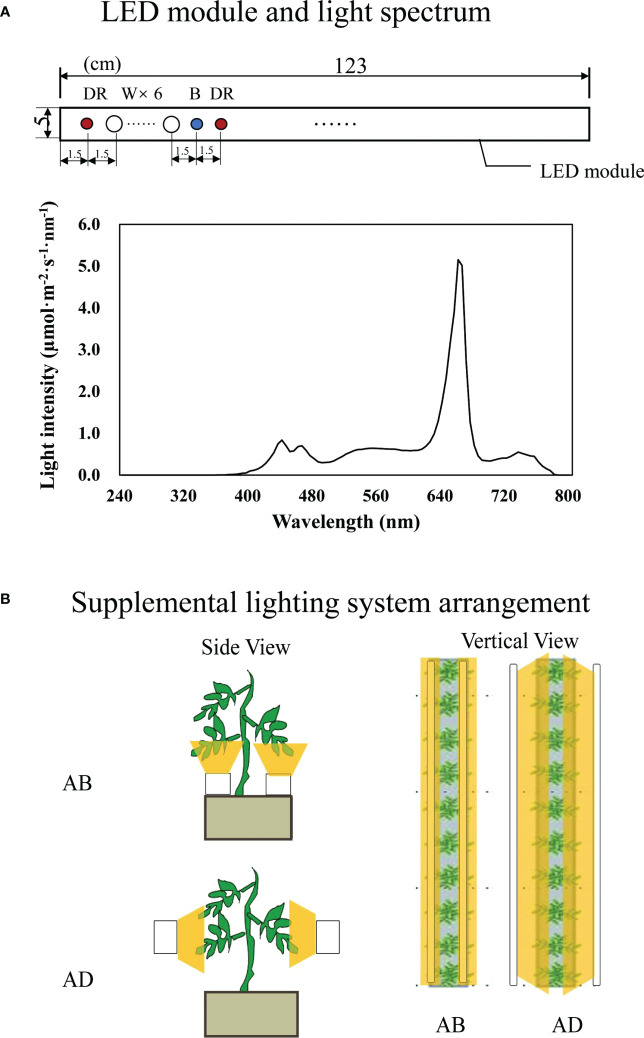
Schematic diagram of LED module characters **(A)** and the supplemental lighting arrangement **(B)**. Abaxial leafy supplemental lighting (AB) and the adaxial leafy supplemental lighting (AD) was applied to plants from the 14^th^ day after transplanting. The supplemental lighting is powered by deep red, white and blue (DR/W/B) LEDs. Each LED module is at a size of 123×5cm and contains 9 groups of color chips (diameter of 7 mm with axis arrangement) at 1DR+6W+1B+1DR **(A)**. LEDs were provided 10 cm from the abaxial or adaxial epidermis of leaves **(B)**, with a PPFD of 200 μmol·m^−2^·s^−1^.

120 tomato seedlings grown in the same environment were taken 2 weeks after transplanting and randomly divided into two groups. Light-emitting diodes (LED; Philips Netherlands Ltd.) were used as light sources (**Figure 1A**). The lighting was processed in two orientations: abaxial leafy supplemental lighting (AB) and adaxial leafy supplemental lighting (AD) (**Figure 1B**), according to previous reports (Song et al., 2016; Jiang et al., 2017) with minor adjustments. The LED was fixed on a movable beam to ensure that illumination distance from the adaxial epidermis of the inner canopy truss or the abaxial epidermis of the lowest leaf truss was maintained at 10 cm. For this, the third leaf from both inner canopy truss or lowest truss was taken as a reference, and plant position was adjusted to ensure vertical growth and a consistent plane of stem axis within the same row when necessary. The supplemental lighting PPFD, measured at a distance of 10 cm from the LED module, was 200 µmol·m^-2^·s^-1^ with a supplemental lighting photocycle of 16h (6:00-22:00), maintained by an integrated digital timer-dimmer-transformer (EEIO-600W-1000W, Shengyuan Electric Appliance Co., Ltd, Zhongshan, China). Each treatment consisted of three rows of plant benches with each row containing 20 plants. After 16 hours of light treatment, the whole leaves of 5 plants from either AB or AD treatment which were randomly selected were mixed as a sample (Liu et al., 2020) for RNA-seq and proteomics analysis.

### 2.3 Gas-exchange parameter measurements

Gas-exchange measurements were conducted based on previous reports (Song et al., 2016). In brief, we selected the second terminal leaflets of leaves on the fifth youngest node with a portable photosynthesis system (Li-6400XT; Li-Cor Inc., Lincoln, NE, USA) during 11:00–16:00, GMT +8 (9:00–14:00, local time) on the 28th day after transplanting. The net photosynthetic rate (*P*_N_), stomatal conductance (*G*s), and transpiration rate (*T*r) were measured. Measurements were conducted with PPFD, leaf temperature, CO_2_ concentration, and relative humidity of 800 ± 5 µmol·m^−2^·s^−1^, 28 ± 1°C, 400 ± 2 µmol·m^−2^, and 63 ± 2%, respectively.

### 2.4 RNA extraction and illumina sequencing

A total of 6 *S. lycopersicum* samples, including 3 AB samples and 3 AD samples, were analyzed by RNA-Seq. Total RNAs were extracted from frozen fresh tomato leaves using an EASYspin Plus Kit according to the manufacturer’s instructions (Aidlab Biotechnologies Co. Ltd., Beijing, China). The quality and quantity of extracted RNAs were measured using agar gel electrophoresis and Nanodrop micro spectrophotometry in combination (Thermo Scientific, Wilmington, DE, USA). RNAs from three biological replicates (0.5 g per sample) across at least five plants with the same concentration and volume were combined for RNA-seq. The sequencing library was constructed using a NEBNext Ultra RNA library prep kit (NEB#E7530, New England Biolabs, Ipswich, MA, USA). The quality of the cDNA library was measured using a DNA 1000 assay Kit (5067-1504, Agilent Technologies, Santa Clara, CA, USA) prior to sequencing on an Illumina HiSeq TM 2500 by Gene *De novo* Biotechnology Co. (Guangzhou, China). RNA-seq data was downloaded from the SRA database (accession number: PRJNA895868). Clean reads were compared to the reference genome sequence using HISAT software (Kim et al., 2015).

### 2.5 Differentially expressed genes (DEGs) analysis

Differentially expressed genes (DEGs) between AB samples and AD samples were identified using the DEGseq software package (http://www.bioconductor.org/packages/2.6/bioc/html/DEGseq.html). Manually identified DEGs (log2 value≥1.5-fold difference, p-value less than 0.01) were then subjected to enrichment analysis using Gene Ontology (GO) functions and KEGG pathways. GO DEGs enrichment analysis provided all GO terms that were significantly enriched in DEGs compared to the genomic background. All DEGs were mapped to GO terms in the Gene Ontology database (http://www.geneontology.org/). Significantly enriched GO terms (FDR correction p-value ≤ 0.05) were identified by a hypergeometric test by comparing them to the genomic background. Pathway enrichment analysis was performed using the Kyoto Encyclopedia of Genes and Genomes (KEGG) database. Pathways with FDR-corrected p-values ≤ 0.05 were defined as significantly enriched DEG pathways.

### 2.6 Protein extraction, iTRAQ labeling, and proteomics analysis

At least 5 seedlings were mixed in each replicate. Total protein was extracted using the cold acetone method and labeled with iTRAQ tags. Shotgun proteomic analyses were performed using an EASYnLC™1200 UHPLC system (Thermo Fisher, Shanghai, China) with an Orbitrap Q Exactive HF-X mass spectrometer (Thermo Fisher, Shanghai, China). iTRAQ quantification was implemented using IQuant software (Wen et al., 2014). Proteins with a fold change of > 1.2 or < 0.8 and unadjusted significance level p < 0.05 were considered differentially expressed proteins.

The enrichment analysis of differentially expressed proteins (DEPs) was performed by GO and KEGG analysis. The iTRAQ proteomic data were deposited in the ProteomeXchange Consortium (http://proteomecentral.proteomexchange.org) *via* the iProX partner repository, using the iProX data license number is PXD038211.

### 2.7 Quantitative real time PCR (qPCR) analysis

A set of DEGs and DEPs identified in this research (30 total genes, 25 genes from RNA-seq and 5 from iTRAQ) were selected were selected for qPCR analysis and verification of transcriptional changes after AB treatments for 0, 1, 2, 4, 6, 8, 12, and 24 hours. AD plants were maintained as controls. Tomato *actin* was used as an internal reference. The primers used were designed using Primer Premier 5.0 (Premier) and are listed in **Table S1**.

### 2.8 Statistical analyses

Triplicate data were analyzed using SAS 9.0 software (SAS Institute Inc., North Carolina, USA) according to SAS Tutorials: Analyzing Data (https://libguides.library.kent.edu/SAS/AnalyzeData). The statistical significance of the difference was evaluated by a Student’s t test and least square means analysis at the level <0.05.

## 3 Results

### 3.1 Phenotypic characterization and gas-exchange parameter

**Figure 2:**
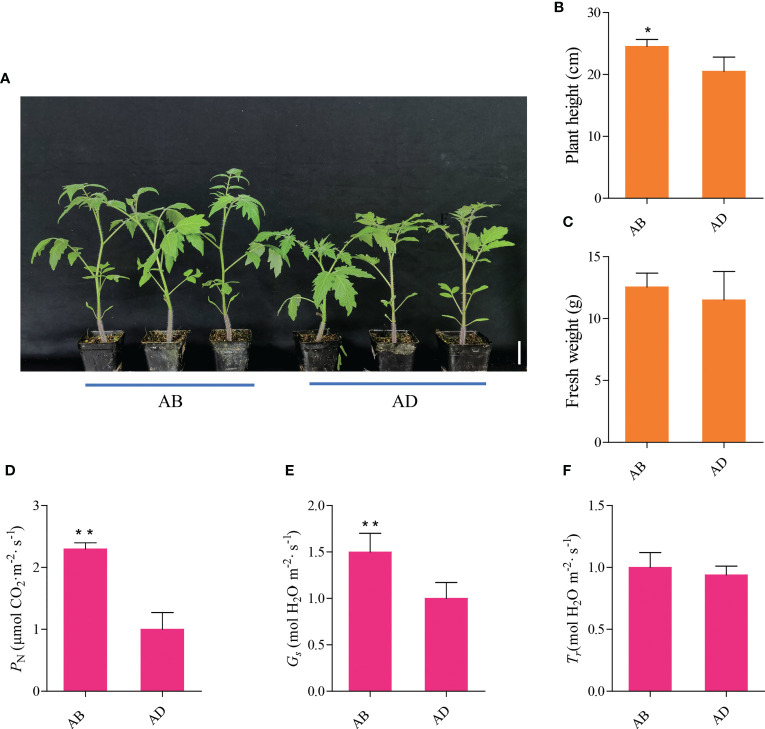
Phenotypic characterization and gas exchange parameters of the abaxial leafy supplemental lighting (AB) and the adaxial leafy supplemental lighting (AD). The ensemble morphology characteristics of plants **(A)**, the effects of AB and AD treatments on plant height **(B)**, fresh weight **(C)**, photosynthetic rate (*P*_N_; **D**), stomatal conductance (*G*s; **E**), and transpiration rates (*T*_r_; **F**) in the leaves of tomato plants. Parameters were measured on the second terminal leaflet of the leaf and the fifth youngest node for each treatment. Measured using PPFD, leaf temperature, CO_2_ concentration, and relative humidity at 800 ± 5 µmol m^-2^ s^-1^, 28 ± 1°C, 400 ± 2 µmol m^-2^, and 63 ± 2%, respectively. Mean ± SE (n = 8). Asterisk indicate significant differences at P < 0.05 according to Student’s t test.

The morphology of tomato seedlings after 2 weeks of the abaxial leafy supplemental lighting (AB) and the adaxial leafy supplemental lighting (AD) can be seen in **Figure 2A**. Compared with AD, AB significantly increased tomato plant height, while fresh weight increased slightly but not significantly (**Figures 2B, C**). In addition, AB significantly increased the *P*_N_ and *G*s (**Figures 2D, E**), while *T*r was increased slightly but not significantly (**Figures 2F**).

### 3.2 Transcriptome sequencing and *de novo* assembly

RNA-Seq analysis yielded an average of 11.23 Gb of data per sample. The average alignment rate of the sample comparison genome was 95.17%, and the average alignment rate of the compared gene set was 88.03%. The number of predicted new genes was 25,970 and the total number of detected expressed genes was 47,249, of which 22,279 were known, and 24,970 were predicted new genes. A total of 33,220 new transcripts were detected, of which 503 belonged to novel alternatively spliced isoforms of known protein-coding genes, and 25,970 belonged to new protein-coding gene transcripts. The remaining 6,747 belonged to long non-coding RNAs.

### 3.3 Gene expression difference analysis

The screening conditions for DEGs were FDR < 0.05 and |log2FC| > 1. A total of 10,998 genes were differentially expressed, with 5280 down-regulated and 5718 up-regulated genes (**Tables S2**). MA plot, Volcano plot, and Scatter-plot were used to display the distribution of DEGs, and an expression heat map was made for each group of DEGs, shown in **Figure S1**.

### 3.4 GO and KEGG enrichment analysis of DEGs

**Figure 3:**
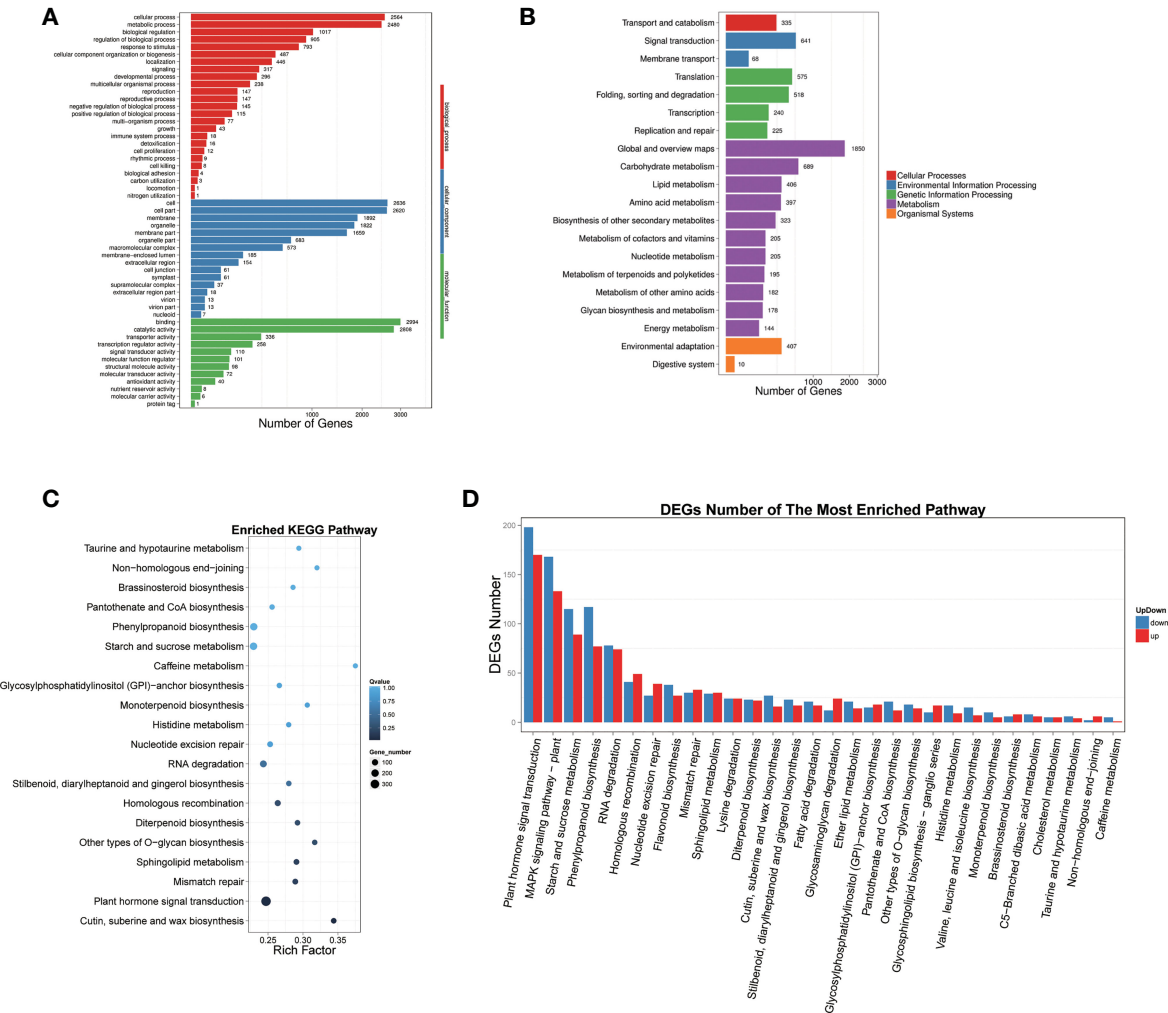
GO and pathway enrichment analyses were performed for all significant DEGs. **(A)** GO classification map of DEGs, the X-axis represents the number of genes, and the Y-axis represents the GO functional classification. **(B)** The X-axis represents the proportion of genes accounted for and the Y-axis represents the KEGG functional classification. **(C)** Pathway enrichment of DEGs, X-axis represents enrichment factor value, Y-axis represents pathway name. The color represents q-value (the whiter the color the larger the value, the bluer the smaller the value), the smaller the value means The smaller the value, the more significant the enrichment result. The size of the dot represents the number of DEGs. **(D)** Enrichment pathways of up- and down-regulated DEGs. The X-axis represents the Pathway entry, and the Y-axis represents the number of up- and down-regulated genes corresponding to the Pathway entry.

GO and pathway enrichment analyses were performed for all significantly DEGs (**Figure 3**). Different comparisons exhibited similar distribution patterns with regard to the numbers and types of enriched pathways, which may be divided into three main functional groups, including 25 biological processes, 16 molecular functions, and 12 cellular components (**Figure 3A**). Significant differences were observed at the level of gene expression in multiple pathways, chiefly including cell process (2564 genes), metabolism process (2480 genes), biological regulation (1017 genes), regulation of biological process (905 genes), response to stimulus (793 genes), and cellular component organization or biogenesis (487 genes).

According to the DEG results, we carried out KEGG biological pathway classification and enrichment analysis. The pathway classification findings illustrated that the functions of DEGs were mainly concentrated in five branches, including Cellular Processes, Environmental Information Processing, Genetic Information Processing, Metabolism, and Organic Systems (**Figure 2B**). The majority of gene functions were focused in metabolic pathways. Pathway enrichment results showed that the top five DEGs were predominantly concentrated in plant hormone signal transduction, cutin, suberine and wax biosynthesis, mismatch repair, sphingolipid metabolism, and other types of O-glycan biosynthesis (**Figure 3C**). Further analysis demonstrated that the DEGs could be classified into 30 categories, and the top five groups were plant hormone signal transduction, MAPK signaling pathway, starch and sucrose metabolism, and phenylpropanoid biosynthesis (**Figure 3D**).

### 3.5 Differentially expressed transcription factor (TFs) and specific regulated genes

We made predictions for DEGs with the ability to encode transcription factors (TFs), and, classified and counted transcription factor families to which the differently expressed genes belonged. Our findings showed that the six most abundant transcription factors were MYB, MYB-related, bHLH, AP2, MADS, and NAC which contained 486, 380, 274, 264, 169, and 155 transcription factors, respectively (**Figure S2**).

We also found that many plant hormone signal-regulated genes were significantly upregulated upon AB treatment, including three gibberellin biosynthesis genes *20ox-3*, *GA2ox5*, and *GAI*, four auxin transport genes *PIN4*, *PIN6*, *PIN7*, and *PIN9*, two auxin response genes *IAA13* and *IAA23*, five auxin response factors *ARF5*, *ARF8*, *ARF9*, *ARF12*, and *ARF18*. Special regulatory genes, such as three sugar transporters SWEET1, SWEET12, and SWEET14, four cell cycle regulators cdkB2, CycA1, CycA2, and cycd3c3, were also significantly upregulated under AB treatment (**Table S2**).

### 3.6 iTRAQ analysis reveals AB responses of tomato leaf proteins

To investigate the effect of AB on protein expression, we performed iTRAQ analysis on tomato leaves under AB and AD treatments. Across all samples, a total of 338,790 secondary spectra were generated and downloaded. Using the filter standard of “1% FDR”, a total of 21,668 peptides and 5,390 proteins were identified. The significantly different proteins (DEPs) were identified as having a fold change> 1.2 or <0.8 and a Q-value < 0.05, and 152 DEPs, including 65 upregulated proteins and 87 downregulated proteins, were identified (**Table S3**).

**Figure 4:**
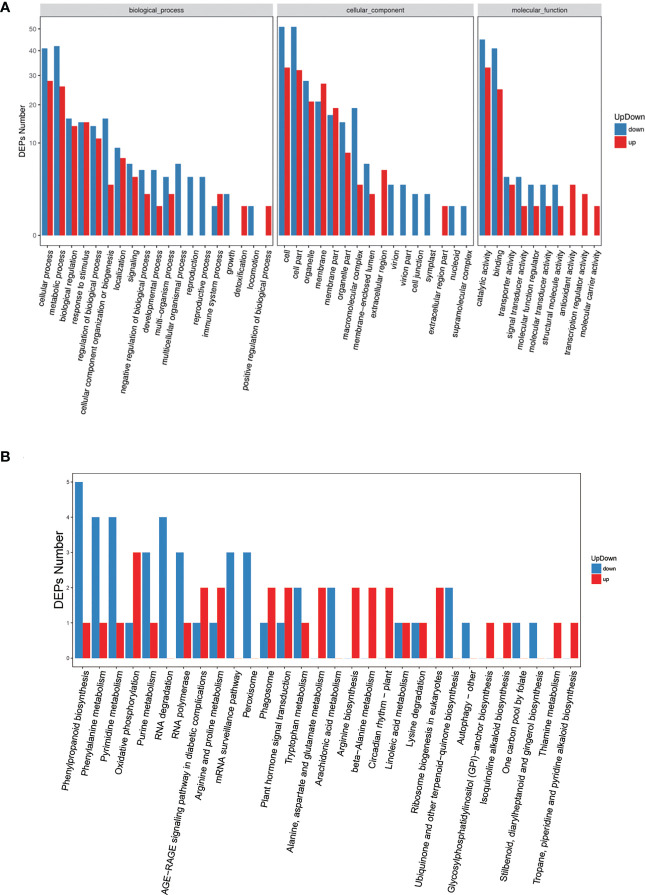
GO and pathway enrichment analyses for all significant DEPs. **(A)** Gene ontology analysis of DEPs, x-axis displays GO term, y-axis displays protein count。**(B)** Pathway analysis of DEPs, x-axis displays pathway name, y-axis displays DEP count.

We further conducted functional classification and enrichment analyses of these identified DEPs. The biological processes of DEPs often included cellular process, metabolic process, biological regulation, response to stimulus, regulation of biological process, and cellular component organization or biogenesis (**Figure 4A**), which was consistent with GO analysis results of DEGs (**Table S2**). Pathway enrichment analysis of DEPs demonstrated that the altered biological pathways were mainly distributed in oxidative phosphorylation, AGE−RAGE signaling pathway in diabetic complications, arginine and proline metabolism, mRNA surveillance pathway, phagosome, and plant hormone signal transduction, et al. (**Figure 4B**).

### 3.7 Integration of transcriptomic and proteomic data

Integrating proteomic and comprehensive transcriptomic data analysis provided an important validation tool for the expression of key genes. However, it has been established that changes in gene expression do not imply a corresponding change in protein content. We integrated transcriptomic and proteomic data to analyze the regulation of gene expression changes in response to protein expression changes. Our findings indicated that 17 genes and proteins were upregulated together, including NADH dehydrogenase, aspartate aminotransferase 3 (ASP3), casein kinase II subunit alpha, stromal cell-derived factor 2-like protein (SDF), protein argonaute 5 (AGO5), xyloglucan endotransglucosylase/hydrolase 1(XTH1), putative ABC1 protein, metal transporter Nramp3 (NRAMP3), PLAT domain-containing protein 1 (PLAT1), lysophospholipase BODYGUARD 3 (BDG3), formate dehydrogenase (FDH1), thiamine pyrophosphokinase 1 (TPK1), inositol phosphorylceramide glucuronosyl transferase 1 (IPUT1), patatin-like protein 1(PLP1), LysM domain receptor-like kinase 3 (LYK3), Non-specific lipid-transfer protein 2 (LE16), and heat stress transcription factor A-1 (HSFA1) (**Table S3**).

Moreover, 18 genes and proteins were downregulated simultaneously, including phenylalanine ammonia-lyase (PAL5), pre-mRNA-splicing factor ATP-dependent RNA helicase (DEAH4), histone H2A.1, serine/threonine-protein kinase (SAPK3), DNA-directed RNA polymerases II, IV, and V subunit 9A (NRPB9A), KH domain-containing protein, protein SMAX1-LIKE 3 (SMXL3), 40S ribosomal protein S16 (RPS16), receptor-like protein kinase (HSL1), polyprenol reductase 2 (PPRD2), basic blue protein, nuclear pore complex protein (NUP1), eukaryotic translation initiation factor 3 subunit H (TIF3H1), histone H2A.1, Chlorophyllase-2 (CLH2), exocyst complex component (EXO70A1), and transcription termination factor (MTERF8).

### 3.8 Verification of RNA-seq using qPCR

**Figure 5:**
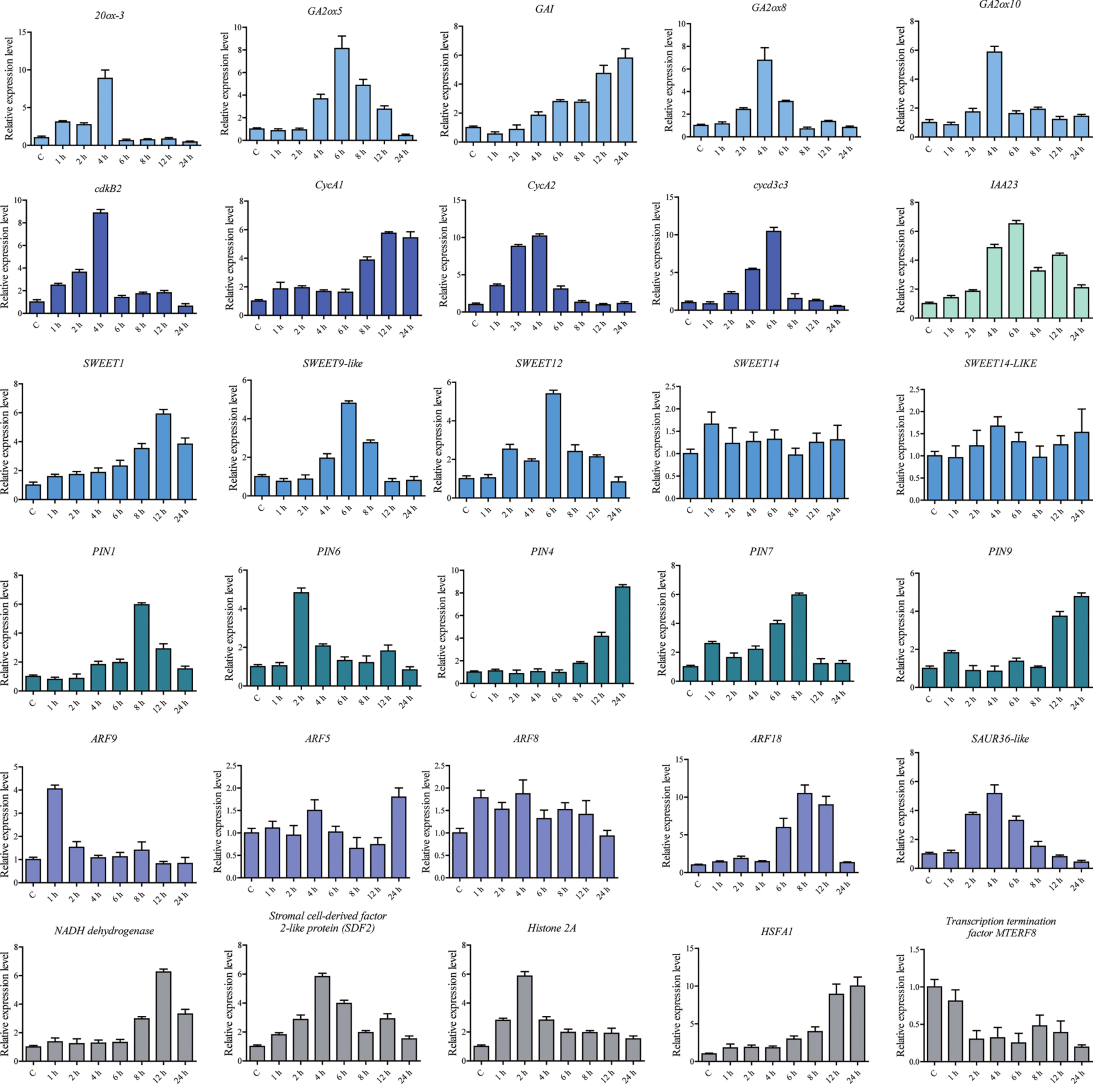
Relative expression levels of DEGs and DEPs analyzed using qPCR under the abaxial leafy supplemental lighting (AB) treatment. The relative expression level of each gene was calculated relative to the expression in the respective untreated control samples (0 h). *Solanum lycopersicum* Sly-*Actin* (*Solyc11g005330.2*) was used as an internal control to normalize the expression data. Different colors represent genes in different signaling pathways. The error bars represent the standard deviation calculated based on three biological replicates.

We used qPCR to further determine gene expression levels in *S. lycopersicum* under AB treatment. The results were consistent with those from RNA-seq and proteomic analyses (**Figure 5**). It was shown that AB does promote upregulated expression of some key genes, such as *20ox-3*, *GA2ox5*, *GA2ox8*, *GA2ox10*, *GAI*, *IAA13*, *IAA23*, *PIN4*, *PIN6*, *PIN7*, *PIN9*, *SWEET12*, *SWEET14*, *CycA1*, *CycA2*, and *Cryptochrome DASH*. Our results ultimately contribute to the understanding of plant light response mechanisms and the discovery of efficient light utilization genes.

## 4 Discussion

Environmental factors can directly alter plant morphogenesis which is theoretically believed to be controlled solely by genetic factors (Wahidin et al., 2013; Song et al., 2016; Jiang et al., 2022). Supplemental lighting has been demonstrated to significantly improve plant photosynthetic performance, increase the biomass and yield of tomato plants, accelerate fruit ripening, improve later fruit quality, and make fruit size and color uniform (Seginer et al., 2006; Song et al., 2016; Jiang et al., 2017). In this study, we observed a significantly increased number of *P*_N_, and *G*_s_ (**Figures 2D, E**), and a slightly but not significantly increased *T*_r_ in plants treated with AB (**Figures 2F**). This was largely consistent with previous research results (Song et al., 2016; Jiang et al., 2017). These results reconfirmed the feasibility of using AB to significantly improve photosynthetic efficiency of a plant to obtain higher economic benefits. From another perspective, this also demonstrated that in-depth analysis of the molecular regulation mechanism of AB treatment on plants bridges the gap between theory and practice for functional gene mining. Additionally, our approach has helped in overcoming or alleviating the damage of light insufficiency stress in greenhouse vegetable production, improving facility industry income, and realizing efficient photosynthetic breeding of crops.

In our study, phenotypic characterization was performed and gas exchange parameters were measured in tomato seedlings under AB and AD treatments, and the results were generally consistent with previous reports (Song et al., 2016; Jiang et al., 2017). However, there was a slight but not significant increase in *Tr*, which may have been related to the node of measurement. Subsequently, we assessed transcriptomic and proteomic changes in tomato leaves under two light supplementation methods by RNA-seq and iTRAQ, respectively. The results illustrated that there were 7,352 differentially expressed genes (DEGs) and 152 differentially expressed proteins (DEPs) between AB and AD treatments. The functions of these DEGs were mainly concentrated in five branches, including cellular processes, environmental information processing, genetic information processing, metabolism, and organic systems (**Figures 3A, B**). Pathway enrichment results demonstrated that the top five DEGs were mainly concentrated in keratin, folinic acid and wax biosynthesis, phytohormone signaling, mismatch repair, sphingolipid metabolism, and other types of O-glycan biosynthesis (**Figure 3C**). Further experiments revealed that DEGs can be divided into 30 categories, with the five most abundant being phytohormone signaling, MAPK signaling pathway, starch and sucrose metabolism, and phenylalanine biosynthesis (**Figure 3D**). Our previous study demonstrated that AB resulted in 15. 8% higher quantum yield of PSII electron transport (ФPSII), 10.2% higher stomatal conductance (*Gs*), 8.5% higher CO_2_ carboxylation efficiency (CE), 10.7% higher tomato fruit yield, and 13.5% higher fruit soluble solids content compared with AD (Song et al., 2016; Jiang et al., 2017). These findings suggest that the differential expression of these genes under AB treatment may be the main reason for the changes in physiological indicators of tomato plants.

Light is an important environmental signal responsible for regulating various growth and developmental processes in plants. Among these light-regulated processes, multiple hormone pathways are commonly regulated by light to mediate developmental changes, such as gibberellin (GA), abscisic acid (ABA), growth hormone, and cytokinin (Jiao et al., 2007; Lau and Deng, 2010). In our study, many phytohormone signaling regulatory genes were significantly upregulated under AB treatment, including three gibberellin biosynthesis genes *20ox-3*, *GA2ox5*, and *GAI*, four auxin transporter genes *PIN4*, *PIN6*, *PIN7*, and *PIN9*, two auxin response genes *IAA13* and *IAA23*, and five auxin response factors *ARF5*, *ARF8*, *ARF9*, *ARF12*, and *ARF18*. In addition, we found specific regulatory genes, such as three sugar transporters *SWEET1*, *SWEET12*, and *SWEET14*, four cell cycle regulators *cdkB2*, *CycA1*, *CycA2*, and *cycd3c3* were significantly upregulated under AB treatment, suggesting that these genes may be related to plant light response.

Integrating proteomic and transcriptomic data revealed that 17 genes were upregulated and 18 were downregulated simultaneously with proteins. These include several key genes that promote plant photosensitivity, enhance photosynthesis, increase plant adaptability, and promote fruit ripening, suggesting that these genes possess potential light-regulated functionality which requires further validation. Notably, many key genes within the mTOR pathway were significantly upregulated (**Figure S3**). In mammals, mammalian target of rapamycin (mTOR), a highly conserved serine threonine protein kinase, is a component of the phosphatidylinositol 3-kinase (PI3K) cell survival pathway which monitors nutrient availability, mitogenic signals, as well as cellular energy and oxygen levels, and is therefore important in regulating cell growth and proliferation (Tsang et al., 2007; Zarogoulidis et al., 2014). mTOR primarily responds to growth factor stimulation and regulates cytoskeletal organization and metabolism. This protein achieves its regulatory effects on cell growth, cell cycle, and other physiological functions mainly through the PI3K/Akt/mTOR pathway, indicating that AB treatment may impact plant development by regulating key genes within the plant mTOR pathway.

To determine the expression levels of some genes under AB treatment, qPCR was performed, and it was determined that AB treatment did promote upregulated expression of some key genes, including *20ox-3*, *GA2ox5*, *GA2ox8*, *GA2ox10*, *GAI*, *IAA13*, *IAA23*, *PIN4*, *PIN6*, *PIN7*, *PIN9*, *SWEET12*, *SWEET14*, *CycA1*, *CycA2*, and *Cryptochrome DASH*. Ultimately, our findings will contribute to a more complete understanding of plant light response mechanisms and to the discovery of genes which contribute to efficient light energy utilization.

## Data availability statement

The datasets presented in this study can be found in online repositories. The names of the repository/repositories and accession number(s) can be found in the article/**Supplementary Material**.

## Author contributions

CJ, JL, and YS conceived and coordinated the project; CJ and JL designed experiments, edited the manuscript, analyzed data, and wrote the first draft of the manuscript; HW analyzed data and performed experiments; XZ and YL provided analytical tools and managed reagents; JW and YZ contributed valuable discussions. All authors contributed to the article and approved the submitted version.

## Supplementary material

Supplementary Figure 1 Statistical results of differentially expressed genes (DEGs). **(A)** MA plot; **(B)** Volcano plot; **(C)** Scatter-plot; **(D)** The expression heat map was made for each group of DEGs. Red and blue points represent up- and down-regulated genes, respectively. Gray points represent no difference genes.

Supplementary Figure 2 Classification of transcription factor (TF) families to which DEGs belong.

Supplementary Figure 3 Differentially expressed genes (DEGs) in the mammalian target of rapamycin (mTOR) pathway.
