# Supplementary figures and images for: Integrating omics reveals insights into tomato abaxial/adaxial leafy supplemental lighting
Source: Front Plant Sci. 2023 Apr 5;14:1118895. doi: 10.3389/fpls.2023.1118895 (PMC10113477; doi:10.3389/fpls.2023.1118895)

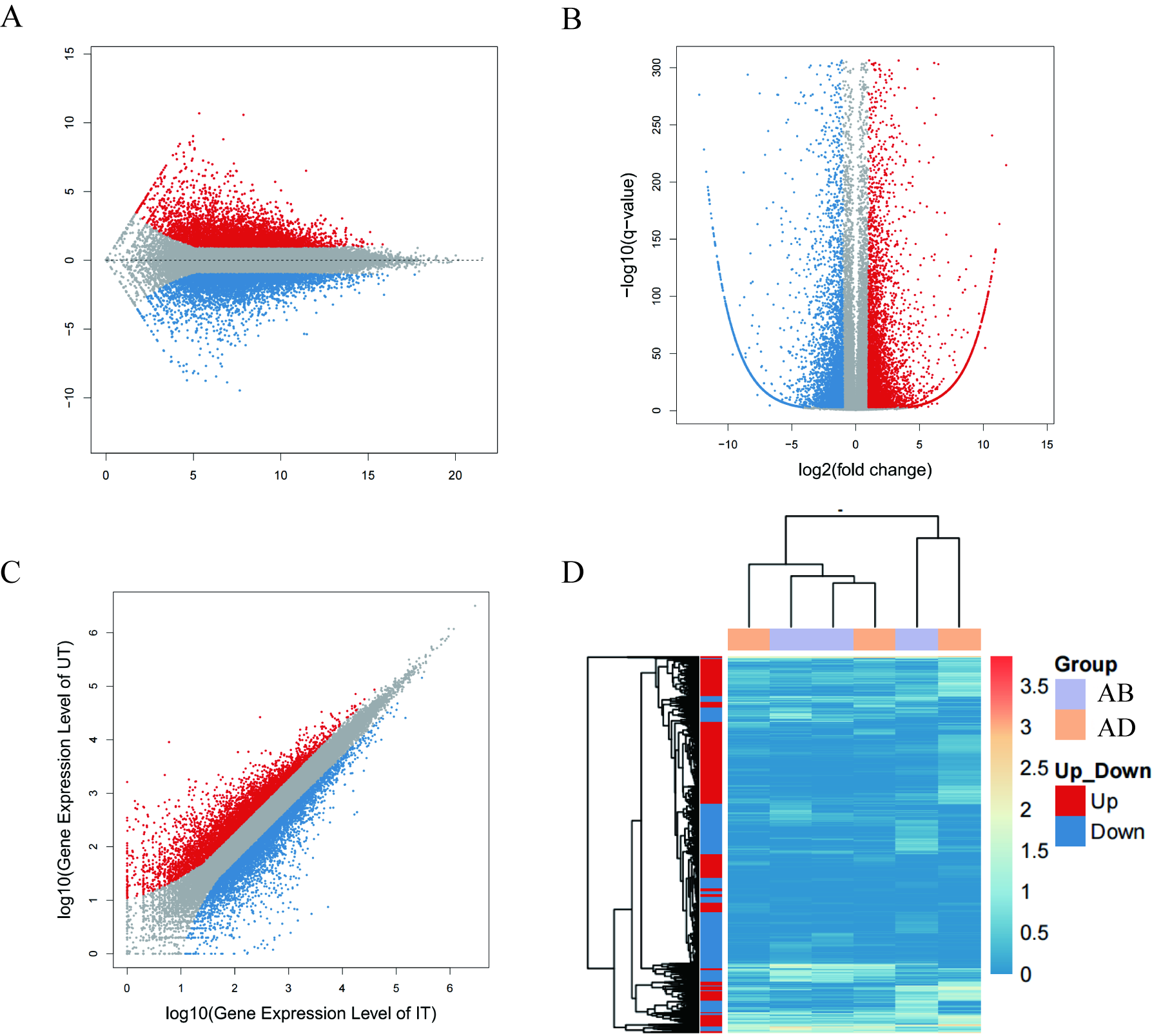

Supplement: Supplementary Figure 1 — Statistical results of differentially expressed genes (DEGs). (A) MA plot; (B) Volcano plot; (C) Scatter-plot; (D) The expression heat map was made for each group of DEGs. Red and blue points represent up- and down-regulated genes, respectively. Gray points represent no difference genes. [file Image_1.tif]

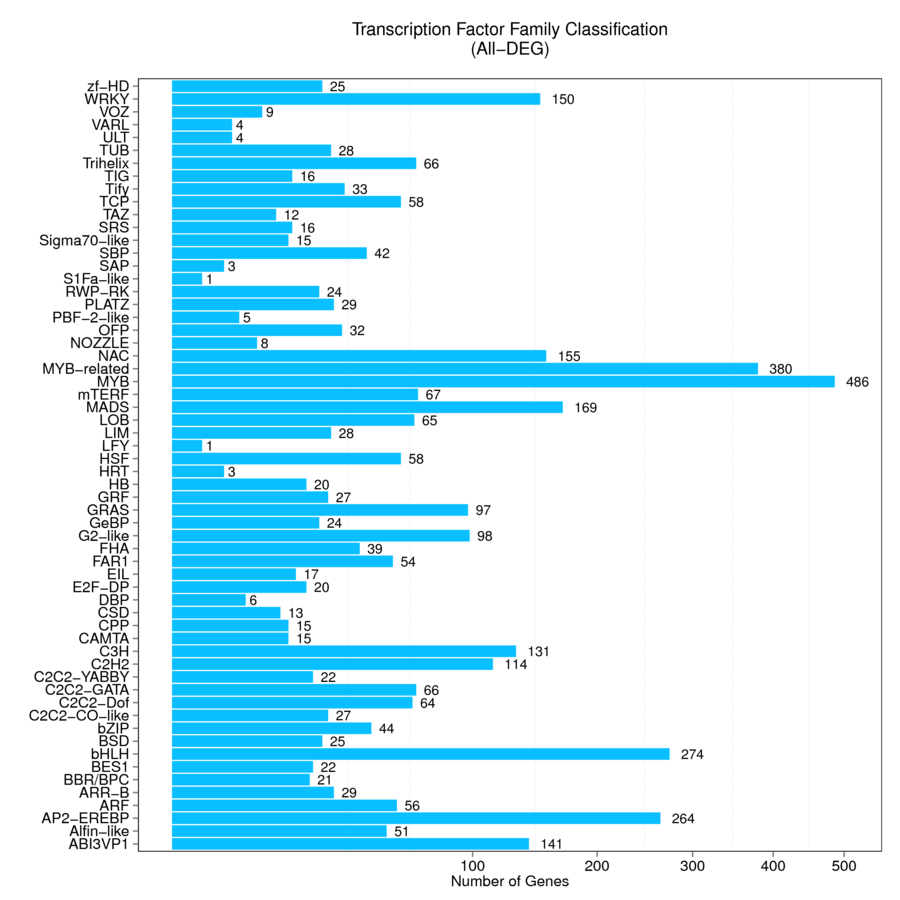

Supplement: Supplementary Figure 2 — Classification of transcription factor (TF) families to which DEGs belong. [file Image_2.png]

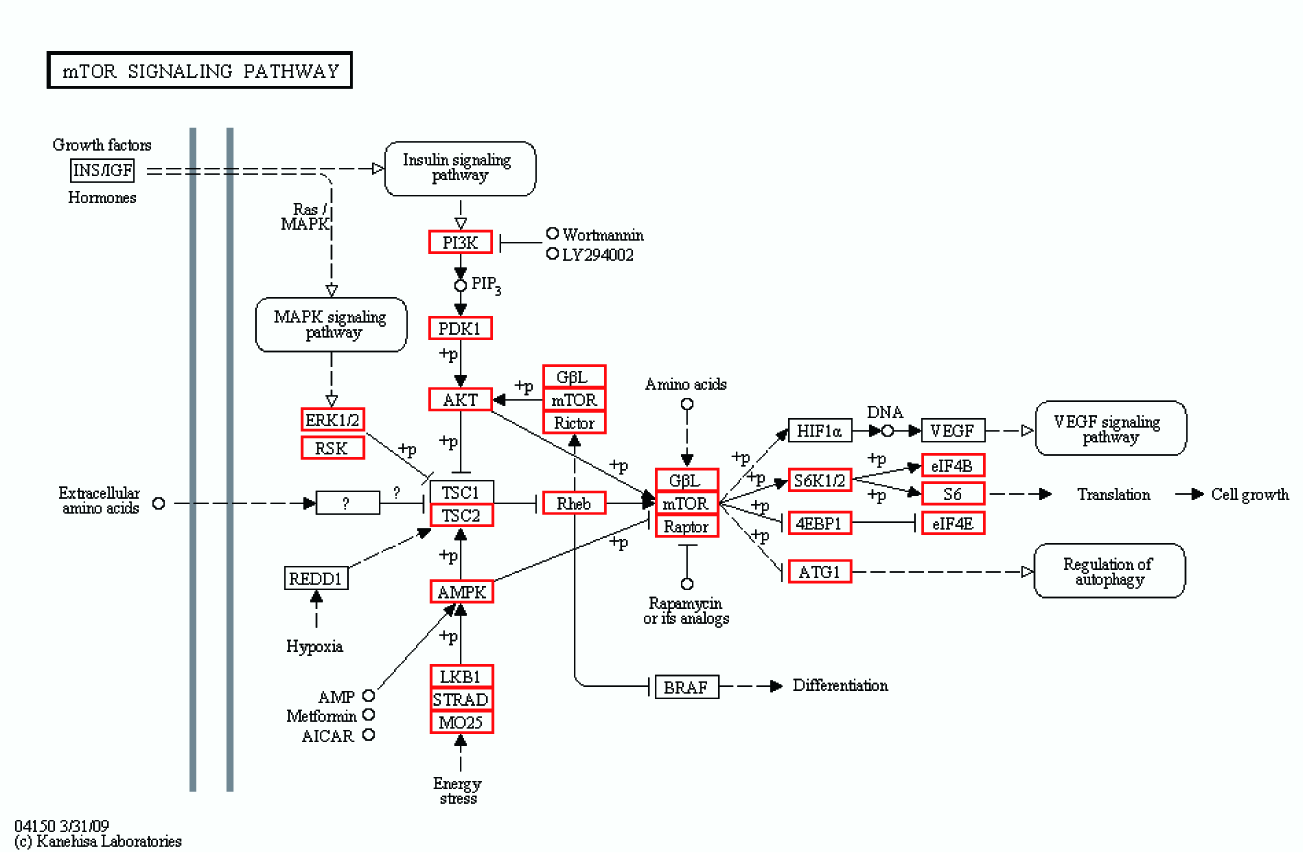

Supplement: Supplementary Figure 3 — Differentially expressed genes (DEGs) in the mammalian target of rapamycin (mTOR) pathway. [file Image_3.tif]
