# Supplementary material for: Integrating omics reveals insights into tomato abaxial/adaxial leafy supplemental lighting
Source: Front Plant Sci. 2023 Apr 5;14:1118895. doi: 10.3389/fpls.2023.1118895 (PMC10113477; doi:10.3389/fpls.2023.1118895)
Supplement: Supplementary file 4 [file Table_1.docx]

**Supplemental Table S1**. Primer sequences used in the study.

| **Gene** | **Sequences** | **AGI number** |
| --- | --- | --- |
| **For quantitative RT-PCR** | |  |
| RT-20ox-3F | 5' TATGCTAGTAGCTTTGTTGGAAGG 3' | Solyc11g072310.2 |
| RT-20ox-3R | 5' GATGGAGAATGGTTAATGATGTGG 3' |  |
| RT-GA2ox5F | 5' CAAATTCTTCTCCTCTCCCCTCT 3' | Solyc07g061730.3 |
| RT-GA2ox5R | 5' AGCTTACTGAAAACATTCGTCGG 3' |  |
| RT-GAIF | 5' CAGGGAAAAGTAAGATTTGGGAAG 3' | Solyc11g011260.1 |
| RT-GAIR | 5' CACCATAGATGAGGATGTAGTTGAA 3' |  |
| RT-GA2ox8F | 5' AGAAAGAATCCGAAGCAGACAAG 3' | Solyc04g008680.3 |
| RT-GA2ox8R | 5' GAAAAGTAAAGAGACGAGAACCCA 3' |  |
| RT-GA2ox10F | 5' CGCTTCGTGTACTGCTTCTTTT 3' | Solyc03g113910.3 |
| RT-GA2ox10R | 5' GTTTTTAACTCCGCCACTTTGA 3' |  |
| RT-cdkB2F | 5' TCCGTGCAAAAGGAGAAACTAT 3' | Solyc04g082840.3 |
| RT-cdkB2R | 5' CTTCATCAAGACCAGGGACAAC 3' |  |
| RT-CycA1F | 5' AACTCGGTTTCTCATTCCTCCA 3' | Solyc11g005090.2 |
| RT-CycA1R | 5' CCAAGTCCACAAGTACATCTCTCTT 3' |  |
| RT-CycA2F | 5' TACCCTCCAGACTACAACACCA 3' | Solyc06g065680.3 |
| RT-CycA2R | 5' TCAGATACCTCCACAAGCCAAT 3' |  |
| RT-cycd3c3F | 5' GTTCCTCTGCTTCTTGACCTTC 3' | Solyc04g078470.3 |
| RT-cycd3c3R | 5' GCCACTTCTTGTATTAGCCTGT 3' |  |
| RT-IAA23F | 5' TCTTCACCCTTTACTTCTTCCC 3' | Solyc04g054280.2 |
| RT-IAA23F | 5' GTTGTGGCTTCATCATCTTCAT 3' |  |
| RT-SWEET1F | 5' TCTTATCATTTGCCGCTATCCT 3' | Solyc06g060580.2 |
| RT-SWEET1R | 5' GGTCCTCCGTTTTAACTTGTCT 3' |  |
| RT-SWEET9-likeF | 5' ATCTCGTTCATTGTGTTCCTTTCTC 3' | Solyc03g097580.3 |
| RT-SWEET9-likeR | 5' TTGGACCCTGTCTTTCTTTGGT 3' |  |
| RT-SWEET12F | 5' TTGTGAGGCTTGCTGGTTTACT 3' | Solyc03g097580.3 |
| RT-SWEET12R | 5' GTTTGGACCCTGTCTTTTTTTG 3' |  |
| RT-SWEET14F | 5' GGTTGGGTGTATTTGCCTTATT 3' | Solyc06g072620.3 |
| RT-SWEET14R | 5' GGCTCTTCCTCTTTCTTGTTGTAG 3' |  |
| RT-SWEET14-LIKEF | 5' TTATTAGTGGTTGGTGGCTTTG 3' | Solyc06g072620.3 |
| RT-SWEET14-LIKER | 5' GGCTCTTCCTCTTTCTTGTTGT 3' |  |
| RT-PIN1F | 5' CATCAAGAAATCCAACACCAAG 3' | Solyc03g118740.3 |
| RT-PIN1R | 5' CTCCAAAAACATCAGAAACAGG 3' |  |
| RT-PIN4F | 5' AGGTCTAGTTCAGAGTTCCCCG 3' | Solyc05g008060.3 |
| RT-PIN4R | 5' TCGAATTTCTTTAGCTCCATCG 3' |  |
| RT-PIN6F | 5' GAATCCGCATTTTCATCCTCTA 3' | Solyc06g059730.2 |
| RT-PIN6R | 5' CCTTTTCTCCAACATCATCTCTG 3' |  |
| RT-PIN7F | 5' CTGCTGCTAGTGTGATGACCAG 3' | Solyc10g080880.2 |
| RT-PIN7R | 5' GCAAAGACGAAAGGAACGATTC 3' |  |
| RT-PIN9F | 5' TTGTTTGGAGTTCAAGTGCTTC 3' | Solyc10g078370.2 |
| RT-PIN9R | 5' TTGGGTTCCTGATTAGTTTTCG 3' |  |
| RT-ARF5F | 5' TCAGAGTTTGTCATTCCTTTGG 3' | Solyc04g081235.1 |
| RT-ARF5R | 5' GGAGGTCTTATCAGCATCTTCAT 3' |  |
| RT-ARF8F | 5' ACTGTCGGAAGTCGAGTTGTTT 3' | Solyc02g037530.3 |
| RT-ARF8R | 5' ATTAGTTCTTGGGCAGGTGGTG 3' |  |
| RT-ARF9F | 5' CAGAGAGAGCACCTCCAAACAC 3' | Solyc08g082630.3 |
| RT-ARF9R | 5' ACGGATAATCACCCATAAGCAT 3' |  |
| RT-ARF18F | 5' TTCTCAGTGGGTATGCGTTTCA 3' | Solyc01g096070.3 |
| RT-ARF18R | 5' ACGGGGTCTTTTGCTCTTTATT 3' |  |
| RT-SAUR36-likeF | 5' AGGAGAAAAAAGGGCTACAAAC 3' | Solyc08g007435.1 |
| RT-SAUR36-likeR | 5' CACAGGGACTAAAACTCTACATGC 3' |  |
| RT-NADH dehydrogenaseF | 5' GAGCCTTGTATCGATCGGATAG 3' | Solyc08g078860.3 |
| RT-NADH dehydrogenaseR | 5' AAAGCAGGAAACACATTTGTGG 3' |  |
| RT-SDF2F | 5' CATCGGAGATTGGAAAGTAGGA 3' | Solyc03g117020.3 |
| RT-SDF2R | 5' CCAGGGTGAGAAAAAGAAAAAG 3' |  |
| RT-Histone H2AF | 5' CTGGTGCTCCTATTTATCTCGC 3' | Solyc01g099410.2.1 |
| RT-Histone H2AR | 5' TGATTCTGCTCTTCTTGTTGTCTC 3' |  |
| RT-HSFA1F | 5' TTGGAATAGAGGAAGAAGTGGA 3' | Solyc03g097120.3 |
| RT-HSFA1R | 5' CCTAGAGAATTGTTAGGCGGGA 3' |  |
| RT-MTERF8F | 5' TGGTGGTGGTGATTTGATTCTT 3' | Solyc03g081300.3 |
| RT-MTERF8R | 5' ATTTTTGCAGCTAGTCCTGGTA 3' |  |
| RT-*Actin*F | 5' TGTGTTGGACTCTGGTGATGGTGT 3' | Solyc11g005330.2 |
| RT-*Actin*R | 5' ATCCAAACGAAGAATGGCATGCGG 3' |  |
